# Pneumonia, Aspiration Pneumonia, or Frailty-Associated Pneumonia?

**DOI:** 10.3390/geriatrics7050115

**Published:** 2022-10-18

**Authors:** David G. Smithard, Yuki Yoshimatsu

**Affiliations:** 1 Queen Elizabeth Hospital, Lewisham and Greenwich NHS Trust, London SE19 4QH, UK; 2 Centre for Exercise, Activity and Rehabilitation, University of Greenwich Southwood Site, London SE9 2UG, UK

**Keywords:** frailty, pneumonia, aspiration, frailty-associated pneumonia, dysphagia

## Abstract

Pneumonia is a common reason for admission afflicting frail older adults. Those who are the frailest are more likely to be provided with a diagnosis of aspiration pneumonia. This diagnosis has no clear definition and no clinical consensus. It is therefore time to stop attempting to differentiate between pneumonia type and use the term frailty-associated pneumonia.

## 1. Introduction

The population in many countries is ageing, with the fastest growing cohort being very old (>80 years of age), who are projected to make up 12.3% of the world’s population by 2029 [1]. The ideal situation is for people to age well with minimal disability, maintaining functioning independence for as long as possible [2]. However, for many, increasing age is associated with increasing dependency and vulnerability. Geriatric syndromes (or giants) are medical problems that frequently present in older adults. They all have multiple aetiological factors and ultimately lead to physical decline and death. In recent years frailty and dysphagia have been included amongst these geriatric syndromes [3,4].

Pneumonia is a common reason for older people to be admitted to hospital, and the literature suggests that those who are the frailest are more likely to be diagnosed with aspiration pneumonia (AP). This paper has been written not to discuss frailty or sarcopenia, per se, but more so to discuss the interpretation of pneumonia in the context of frailty. We discuss the relationship between pneumonia and frailty and suggest that there should be a change of nomenclature and, rather than trying to differentiate pneumonia “types”, pneumonia in older frail adults should be called frailty-associated pneumonia (FAP).

## 2. Pneumonia

Pneumonia occurs in people of all age groups and is often classified depending on the frailty of the patient, the place of residence at the time of the infection (community, hospital, or ventilator), or its presumed methodological aetiology (e.g., aspiration) [5].

Increasing age, frailty, smoking, immunosuppression, and comorbid conditions are all risk factors for CAP [6]. The aetiological agents of CAP has varied little over the years with Streptococcus pneumoniae being accepted as most common pathogen; others include Staph Aureus, Legionella pneumophilia, Pseudomonas aeruginosa, and Haemophilus influenzae [6,7] and this has changed little over the years.

Between 2002 and 2017, the incidence of clinically diagnosed pneumonia increased from 1.5/1000 person years to 2.22 per 1000 person years [8], with the prevalence being reported as 6 times higher in those >75 years compared to those <60 years of age [9,10]; 164.3 cases/10,000 adults > 80 years of age accounting for 45% of diagnosed CAP occur in people >65 years of age [11]. Hospital-acquired pneumonia occurs in 5–10/1000 admissions [12]. Pneumonia is not a benign problem. CAP has a mortality rate of 2–5/1000 years [13,14]. In 2016, there were approximately 2.3 million deaths from pneumonia [15] and 2.5 million in 2019 [16]. A total of 3.2 million people die from influenza and pneumonia each year [17].

## 3. Nutrition

Good nutrition is pivotal to a healthy life. With increasing age, access to high quality food becomes increasingly difficult, not only because of illness but also because of socio-economic factors. Lack of good quality food in the right quantity coupled with anabolic resistance [18,19] can ultimately result in protein and micronutrient undernutrition. The clinical consequences of this can manifest as frailty, sarcopenia, and an ineffective immune system with an increase in morbidity and eventual death [19,20].

## 4. Frailty and Oral Frailty

Frailty has been classified as a wasting syndrome [21] characterised by weakness and a poor nutritional status [22]. Ferrucci et al. defined frailty as “a physiological syndrome, characterised by decreased reserve and diminished reserve to stressors, resulting from cumulative decline across multiple physiological systems, resulting in multisystem dysregulation and causing vulnerability to adverse outcomes” [23,24].

Fried et al. [21] argued that there is a frailty phenotype presenting with weight loss, exhaustion, low energy expenditure, reduced muscle strength, and slowness in walking [25]. Rockwood, on the other hand, viewed frailty as an accumulation of deficits rather than being a specific phenotype [26]. Many old frail adults have many comorbidities including lung disease, ischaemic heart disease, stroke, dementia, and diabetes.

The prevalence of frailty increases with age, such that one-quarter to one-third of adults >85 years will be frail [27,28] Severity of frailty increases the ability to cope with acute stressors, such as infection [27], and those with severe frailty have a significantly high mortality [26,27]. Hence, a cycle of decline occurs as recovery from any stressor is usually not associated with a return to the previous level of functional ability.

Sarcopenia, which is an intrinsic component of the frailty syndrome [29], not only involves axial muscles but also those involved in chewing (masseter) and swallowing (supra- and infra-hyoid groups of muscles) [30]. Difficulty in chewing has been termed “oral frailty” [31,32].

As people become more dependent on others for personal oral hygiene, there is a deterioration of oral health [32,33,34]. Higher oral bacterial counts are found [35,36], gram negative and anaerobic organisms are increasingly present in the microbiome, and the nature of any infection may change [37]. Studies have shown that, for those most at risk, effective proactive mouth care can mitigate any risk [38,39]. Poor oral health and oral frailty are associated with an increased mortality and morbidity [40].

## 5. Frailty and Immunity

Over the life span, people have multiple and repeated exposure to environmental toxins, resulting in a proinflammatory milieu as demonstrated by an increased white cell count, CRP, chemokine, and cytokines (inflammageing) [23,41]. This results in the expression of senescence-associated molecules [42], and hence a decreased efficiency of the adaptive and innate immune systems [43] or “immunosenescence” [44] with an impaired immune surveillance, a reduced response to antigens [44,45], and an inability to generate immunoreactive T cells [46].

**Figure 1:**
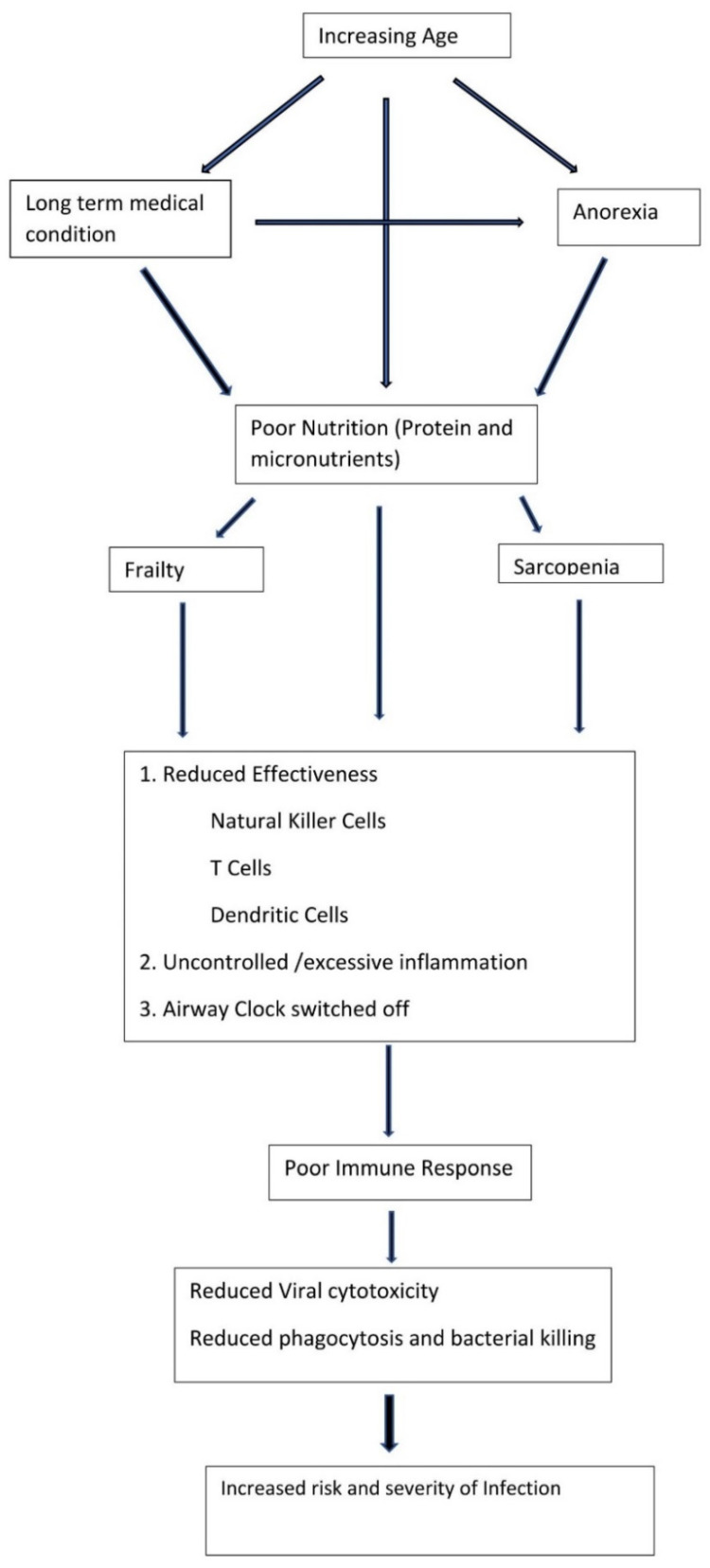
Mechanism of increased infection risk in older frail adults.

In the presence of an infection, there is a maladaptive immune response with a reduced proliferation of mononuclear cells, and an attenuated adaptive immune response with an overexpression of inflammatory agents exacerbating the chronic inflammatory state and a cycle of functional decline [6,23,41]. This maladaptive and poorly effective response to infection (Figure 1) not only results in an increased risk of infection but any infection is likely to be more severe and is more likely to result in a decline in function or death [47].

## 6. Lungs and Immunity

The lungs have their own immunity milieu, which mirrors the systemic immune system, but also includes a resident microbiome [48,49,50,51]. Epithelial cells and alveolar macrophages modulate immunity by production of mucins, cytokines, and tissue necrosis factor [52]. This aspect of lung defence has an internal clock or circadian rhythm, which is important in maintaining the integrity of the lung’s immune response.

The physical defences of the lung include airway patency, a functional mucociliary escalator, and an effective cough. Mucus, produced by goblet cells and submucosal glands (also assisted by Clara cells and tissue fluid transudation) are conveyed into the trachea and larynx by a coordinated wave of ciliary motion of ciliated epithelial cells [53]. This itself can bring mucus up to the oral cavity to be swallowed or spat out.

As people get older and frailer, together with the immunosenescence described earlier, there is a disruption of the airway cell clock or circadian rhythm of mucus production [54], all resulting in an increased susceptibility of older frail adults to endotoxin damage resulting in pneumonia [46,55]. (Figure 1).

The ability to clear the lungs of any organism or foreign body inhaled or aspirated is also impaired. There is a reduction in laryngeal sensation (due to age or neurological disease, resulting in a reduced urge to cough) [44,56] and reduced respiratory muscle strength may result in a reduction in the peak expiratory cough velocity [57,58]. Mucus quality is poor, its immune function degraded, and mucociliary clearance becomes inefficient [59,60,61,62]. Air movement, tidal volume and functional residual capacity are reduced secondary to damaged lung elasticity in association with coexistent lung disease [59,60,61,62]. All of these may result in mucus pooling and increased risk of lung infection.

## 7. Dysphagia

Dysphagia is a common problem for frail older adults [30,63], with 55% of frail adults reporting problems with swallowing [63]. Issues include fatigue whilst chewing (oral frailty), taking longer to eat, and coughing and choking whilst taking medication or eating and drinking. For some, swallowing is intact until they become acutely unwell, then; due to their precarious physiological state, swallowing becomes transiently unsafe whilst they remain unwell. Rofes et al. reported that two-thirds of frail older adults presented with oropharyngeal residue, >50% had laryngeal penetration, and 17% had food and liquid entering the airway [64]. Frail adults who have a swallowing impairment have a higher mortality rate [64] and are more likely to develop pneumonia [65].

Dysphagia is present in many (53–92%) older adults who present with pneumonia [66,67] but is often not sought in the clinical environment [68]. Chojin et al., 2017 found that, of 153 people admitted to hospital with pneumonia (mean age 85.4 ± 9.9 years), 110 (72%) had an abnormal swallow assessment using the MASA (dysphagia and associated risk of aspiration) [69]. The premorbid prevalence of dysphagia in older adults’ people presenting with pneumonia is unknown; some have pre-existing dysphagia and other older people may well have decompensated swallowing as a secondary phenomenon of infection in the context of frailty.

## 8. Pneumonia and Frailty

The label, and hence the medical management, including antimicrobial treatment, given to “pneumonia” has been defined more by environmental considerations over the years rather than by microbiological evidence, hence the labels healthcare-acquired, community-acquired, and aspiration pneumonia. In reality, this separation lacks utility for the admitting physician and may adversely influence the medical care provided [70,71,72].

The aetiology of pneumonia in frail older adults is likely to be multifactorial in nature due to their lifetime exposure to toxins, multiple comorbidities (and polypharmacy), probable swallowing impairments [60,61], and maladaptive immunity [42]. Adults who have marked frailty (Clinical Frailty Score 7/8) with multiple comorbidities and who reside in a nursing home are more likely to be diagnosed with AP than other older adults [65,70,71,73,74,75,76,77].

This diagnosis is often made with little objectivity [10,78] and is dependent on the individual clinician’s subjective impression [79], and there is little documented consensus between clinicians [65].

The general clinical working definition for AP is “the development of pneumonia secondary to the presumed entry of oropharyngeal secretions into the lung, based solely on clinical suspicion and CXR changes with no supportive clinical observational or microbiological evidence” [70,80]. The American and British Thoracic and Respiratory Societies do not have guidelines or definitions of AP [12,81,82]. In Japan, the definition is one of “pneumonia in a patient with a predisposition to aspirate (due to dysphagia or swallowing disorders) and a predisposition to decreased airway clearance and pneumonia (due to a chronic lung disease or being bedbound)” [83].

Aspiration of saliva from the oral cavity into the tracheal-bronchial-alveolar tree via the pharynx is reported to be common but lung defences prevent any adverse outcomes [61]. Amongst older adults who are hospitalized with CAP, aspiration is observed in one-third of patients who are in their 50s, 50% of patients who are in their 60s, >80% of patients who are ≥70 years old [79], and possibly 92% of admitted frail older people [84]. Aspiration of oropharyngeal secretions is not a sine qua non of aspiration pneumonia [66]; Langmore et al. stated “just because someone has swallowing problems and may have aspiration, does not mean that aspiration is the cause of pneumonia” [85].

**Figure 2:**
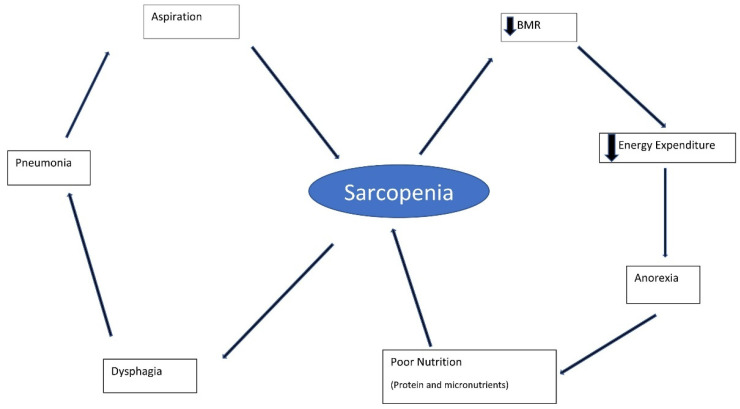
Frailty–pneumonia cycle. BMR: Basal metabolic rate.

Pneumonia associated with aspiration can be the result of repeated aspiration of small amounts of pepsin within saliva [84], causing inflammation combined with reduced immune function [85] and impaired lung function, rather than an acute event. With chronic micro-aspiration and increased vascularity, there is an increase in cytokines (including TNFα and WEGF). Increased cytokine concentrations induce a decrease in muscle mass and strength (sarcopenia), increased frailty, worsening dysphagia, and possible further aspiration [56,84] (Figure 2).

It is likely that the presence of risk factors for aspiration are more important than aspiration, per se [86], with many having more than one comorbidity, and a third may have >3 [87]. Manabe et al., 2015 suggested the following four factors, three of which could have been secondary to rather than aetiological to pneumonia [14]. Older and frail adults with polypathology frequent take many medications and, as a consequence, have a high anticholinergic burden (ACB). Castejon-Hermandez et al., 2021 found that those adults with an ACB ≥ 3 were >4 times more likely to develop dysphagia [88] and hence be at risk of aspiration and its complications [78].

## 9. Is It Time for Frailty-Associated Pneumonia?

Pneumonia commonly occurs following a stroke. In 2003, Hilker et al. coined the phrase stroke-associated pneumonia (SAP) to describe pneumonia occurring after an acute stroke, particularly in those admitted to intensive care units [89]. It has become accepted that SAP covers a spectrum of pulmonary infections [90,91] and that the aetiology of pneumonia after stroke is not purely infective in nature. Following a stroke, there is a transient inhibition of cell-mediated immunity demonstrated with a lymphopenia and monocyte deactivation, alteration of tracheal epithelium, and impaired airway clearance. Researchers studying animals noted that this inhibition resulted in spontaneous systemic bacterial infection within three days of the acute stroke [92]. This is driven by an overactivation of the hypothalamic–pituitary–adrenal axis and activation of the sympathetic nervous system [91,92,93], which has been inhibited by a beta receptor blockade [92].

Clinical risk factors for SAP are accepted to be stroke severity, reduced consciousness level, dysphagia, and the presence of long-term conditions (e.g., lung disease and ischaemic heart disease) [91].

As we have shown earlier, pneumonia occurring in older frail adults is complex and has many similarities to pneumonia in stroke (Table 1). Frail adults have a poorly functioning immune system, reduced airway clearance, altered bronchial epithelium, and multiple long-term conditions (Figure 1).

Given the frequency of aspiration and lack of microbiological differentiation between CAP, HAP, and AP, it is likely that they are all variations of the same clinical entity [76,94,95]. By clinically differentiating them, clinicians might ignore the potential for aspiration [66], potentially causing many people to be under-investigated [70].

In 2018, Ferguson et al. recommended dropping AP due to its lack of clinical accuracy and potential for causing harm to the patient [96]. Therefore, we suggest that it is time to designate pneumonia occurring in old and frail individuals as frailty-associated pneumonia (FAP) rather than CAP or AP.

## Figures and Tables

**Table 1:** Factors implicated in stroke-associated pneumonia and frailty-associated pneumonia.

Acute Stroke (SAP)	Frailty (FAP)
Immune dysregulation	Immune dysregulation
Reduced airway clearance	Reduced airway clearance
Swallowing Impairment	Swallowing Impairment
Recurrent Infection	Recurrent Infection
Multiple comorbidities/long term conditions	Multiple comorbidities/long term conditions
Poor prognosis	Poor prognosis

## Data Availability

Not applicable.
